# Enhanced recovery care versus traditional care following laminoplasty: A retrospective case-cohort study

**DOI:** 10.1097/MD.0000000000013195

**Published:** 2018-11-30

**Authors:** Jun Li, Hao Li, Zheng-kuan Xv, Jian Wang, Qun-fei Yu, Gang Chen, Fang-cai Li, Ying Ren, Qi-xin Chen

**Affiliations:** Department of Orthopedics, the Second Affiliated Hospital, School of Medicine, Zhejiang University, Hangzhou, China.

**Keywords:** cervical, complications, enhanced recovery after surgery, hospitalization, laminoplasty

## Abstract

Enhanced recovery after surgery (ERAS) has been shown to shorten length of hospital stay and reduce perioperative complications in many types of surgeries. However, there has been a paucity of research examining the application of ERAS to major spinal surgery. The current study was performed to compare complications and hospital stay after laminoplasty between an ERAS group and a traditional care group.

The ERAS group included 114 patients who underwent laminoplasty managed with an ERAS protocol between January 2016 and June 2017. The traditional care group included 110 patients, who received traditional perioperative care between November 2014 and December 2015. Postoperative hospital stay (POPH), physiological function, postoperative visual analogue scale (VAS) pain score, and postoperative complications were compared between the 2 groups.

The mean POPH was significantly shorter in the ERAS group than traditional care group (5.75 ± 2.46 vs. 7.67 ± 3.45 d, *P* < .001). ERAS protocol significantly promoted postoperative early food-taking (8.45 ± 2.94 h vs 21.64 ± 2.66 h, *P* < .001), reduced the first time of assisted walking (30.79 ± 14.45 vs. 65.24 ± 25.34 h, *P* < .001), postoperative time of indwelling urinary catheters (24.76 ± 12.34 vs. 53.61 ± 18.16 h, *P* < .001), and wound drainage catheters (43.92 ± 7.14 vs. 48.85 ± 10.10 h, *P* < .001), as compared with the traditional care group. Pain control was better in the ERAS group than traditional care group in terms of mean VAS score (2.72 ± 0.46 vs. 3.35 ± 0.46, *P* < .001) and mean maximum VAS score (3.76 ± 1.12 vs. 4.35 ± 1.15, *P* < .001) in 3 days after surgery. The morbidity rate was 21.05% (24 of 114 patients) in the ERAS group and 20.90% (23 of 110 patients) in the control group (*P* = .75).

The ERAS protocol is both safe and feasible for patients undergoing laminoplasty, and can decrease the length of postoperative hospitalization without increasing the risk of complications.

## 1 Introduction

The enhancing recovery after surgery (ERAS) protocol is an evidence-based multimodal perioperative care approach to reduce the length of hospitalization, diminish surgery-related complications, and improve early rehabilitation. The ERAS protocol, also known as “fast-track surgery” or “enhanced recovery program,” has been successfully applied in abdominal surgery.^[1–4]^ In orthopedic surgery, the ERAS protocol was first adopted in high-volume procedures, such as primary hip and knee arthroplasty, and there is much evidence that the ERAS protocol is both effective and safe.^[5,6]^ However, there has been a paucity of research examining the application of ERAS to major spinal surgery.

The development of an ERAS protocol for use in the Division of Spinal Surgery of our hospital began in 2015. Since the most common spinal surgery is posterior lumbar decompression and fusion with fixation (PLDF), we first establish an ERAS protocol for lumbar surgery.^[7]^ Our previous study showed that the ERAS protocol for PLDF can facilitate the recovery of physiological function and reduce the incidence of postoperative pain, postoperative complication to shorten the period of postoperative hospitalization. The ERAS protocol presents a good model for other spinal surgery procedures.

Laminoplasty is increasingly performed in patients with multilevel cervical spinal cord compression or spinal canal stenosis. As compared to the less invasive anterior cervical approach, the posterior cervical approach is associated with a greater risk of surgical trauma and complications,^[8–10]^ because the posterior muscles and ligamentous structures are widely dissected and the lamina are removed or opened. The posterior cervical approach is also associated with a greater estimated blood loss, longer surgical duration, and hospital stay, as compared to the anterior cervical approach.^[10]^ A recent study.^[10,11]^ A recent study^[11]^ of an American national database of 2613 patients found that the complication rate following cervical laminoplasty was 22.5%, with the most common complications being dysrhythmia, infections, pulmonary issues, paralysis, neurologic complications, hematoma, wound dehiscence, chronic pain, deep vein thrombosis, and pulmonary embolism, which may lead to a longer period of hospitalization after surgery. Thus, establishing an ERAS protocol for cervical laminoplasty is more urgent than for the anterior cervical approach. The aim of this retrospective study was to compare the incidences of complications and length of postoperative hospitalization after laminoplasty between an ERAS group and a traditional care group. To our knowledge, this is the first study to evaluate the effectiveness of an ERAS protocol for laminoplasty.

## 2 Materials and methods

### 2.1 ERAS program

Our ERAS protocol was implemented in the Division of Spinal Surgery of our hospital on January 1, 2016. Based on the ERAS protocol of the Department of Hepatobiliary and Pancreatic Surgery of our hospital,^[12]^ an ERAS protocol for spinal surgery was developed by both doctors and nurses of the Division of Spinal Surgery. The protocol was first implemented for patients undergoing PLDF, which was the most common surgery in our division. The ERAS protocol was found to reduce the risk of adverse events during the perioperative period, promote rehabilitation, and shorten the hospital stay after surgery.

Our ERAS protocol for spinal surgery included the following elements (Table 1): preoperative education, no bowel preparation, antimicrobial prophylaxis, multimodal analgesia, postoperative nausea and vomiting (PONV) prophylaxis, early oral food intake, less infusion volume, antithrombotic prophylaxis, early removal of wound drainage and urinary catheters, early mobilization, and discharge criteria. Preoperative education included the aim and procedure of the ERAS protocol, pain coping strategies, discharge criteria, and a follow-up plan. The fasting guidelines called for the cessation of clear liquids for 2 hours, as well as solid foods for 6 hours before anesthesia.^[13]^ A protocol to maintain intraoperative temperature with the use of short-acting anesthetics and preoperative antibiotics was based on popular guidelines. The temperature of the operation room was controlled at 25°C and warm fluids and upper and lower body air-warming devices are used. Multimodal analgesia included .75% ropivacaine for local anesthesia around the incision, patient-controlled analgesia infusion pump based on patient needs, intravenous nonsteroidal anti-inflammatory drugs (40 mg of parecoxib every 12 h or 100 mg offlurbiprofen (Axetil or Kaifen, b.i.d.) for 3 days after surgery and then sequential oral administration of 100 mg of celecoxib (Celebrex, b.i.d.). Regular diet was given after anesthesia awareness on the day of surgery. A lower infusion volume was defined as less than 1000 mL/d postoperatively. The urinary catheter was removed within 24 hours after surgery. Wound drainage was strongly recommended to be removed within 48 hours after surgery. Early mobilization included on-bed movement on the day of surgery, as well as sitting and assisted walking on postoperative day (POD) 1. The antithrombotic prophylaxis mainly includes preoperative deep venous ultrasound for high-risk patients, postoperative use of a lower extremity pneumatic pump and stretch socks, on-bed movement, and early off-bed mobilization. Discharge criteria were as follows: the patient can walk with help; a visual analogue scale (VAS) score of ≤ 3 points with/without the use of oral analgesia; normal diet, no need for intravenous fluid; normal body temperature, no wound infection; and no severe complications. We encouraged patient discharge once the discharge criteria were met.

**Table 1:**
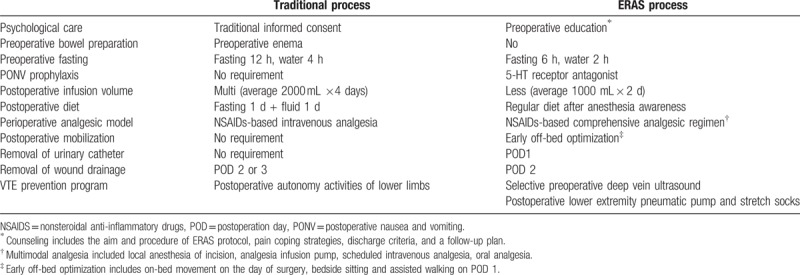
The ERAS protocol versus traditional care.

### 2.2 Populations

The medical records of patients who underwent cervical laminoplasty for degenerative multilevel spine compression and spinal canal stenosis in our spine division were retrospectively reviewed. This study was approved by the Ethics Committee of the Second Affiliated Hospital, School of Medicine, Zhejiang University. The ERAS group consisted of patients who received the ERAS protocol from January 2016 to July 2017. The control group consisted of patients who received the traditional protocol from November 2014 and December 2015. Patients with diseases that may bias the results before surgery, such as neoplastic diseases, severe multiple trauma, cervical fracture, atlantoaxial diseases, and critical cardiopulmonary diseases, were excluded from analysis.

### 2.3 Surgical techniques

**Figure 1:**
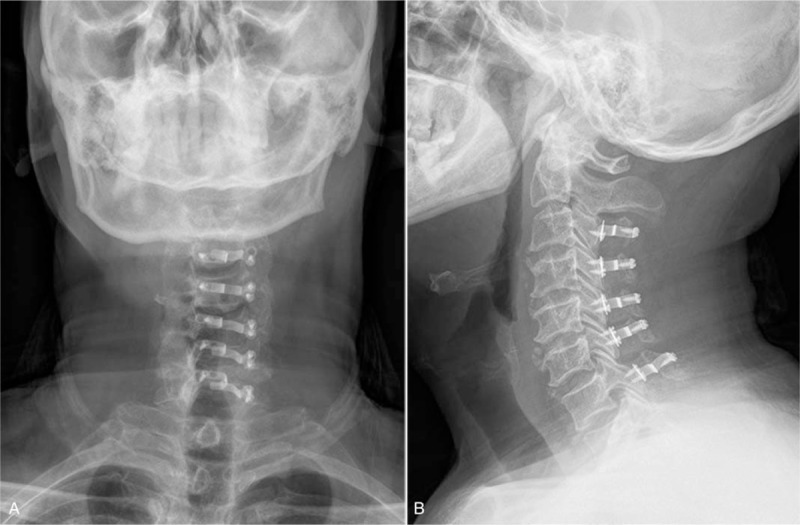
C3–C7 laminoplasty. A, Anteroposterior view. B, Lateral view.

All patients underwent unilateral open-door laminoplasty, which is also called the Hirabayashi technique (Fig. 1). A high-speed drill was used to open the lamina on the right/left side. A shallow trough was drilled on the contralateral lamina, which was used as a hinge to open the lamina. This procedure generally performed at C3– C7 with the use of titanium miniplate to secure the opened laminae. Patients engaged in moderate physical training at POD 1 and recommended to wear Philadelphia cervical collar for 2 weeks.

### 2.4 Clinical evaluation variables

Physiological function outcome indicators, such as the first time that the patient was able to eat, defecate, and get-up, as well as the removal time of the urinary and wound drainage catheters, and discharge time were recorded.

The postoperative pain criteria of 3 days after surgery were recorded, which included the average VAS score, the highest VAS score, and the frequency of break-out pain (VAS score ≥ 5). The Japanese Orthopedic Association (JOA) score was applied to measure the neurological status preoperatively.

The incidences of all postoperative complications and adverse reactions, such as C5 palsy, incisional infection, axial pain, vomiting, neurologic complications, pulmonary infection, urinary tract infection, and deep venous thrombosis, were recorded.

### 2.5 Statistical analysis

Descriptive data are presented as the mean ± standard deviation. Statistical analysis was performed with the independent sample *t*-test, chi-square test, and nonparametric test using SPSS ver. 18.0 software (SPSS Inc, Chicago, IL). A probability (*P*) value of < .05 was considered to indicate statistical significance.

## 3 Results

### 3.1 Demographic data

Demographic patient data and preoperative characteristics are shown in Table 2. There were no significant differences in age, sex, alcohol intake, smoking status, surgical duration, intraoperative blood loss, JOA score, VAS score, and other comorbidities between the traditional care group and the ERAS groups.

**Table 2:**
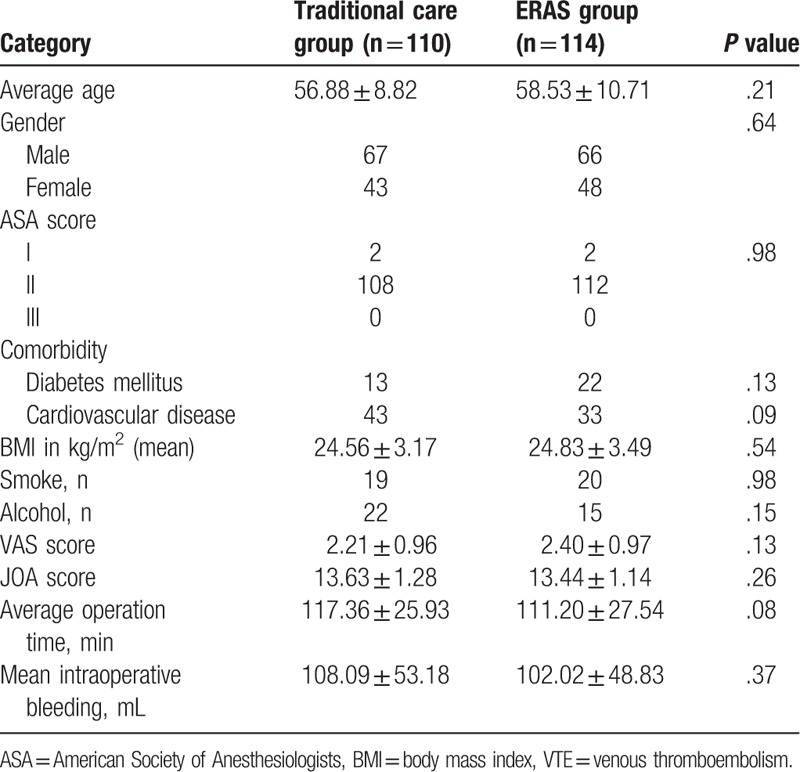
Demographic characteristics.

### 3.2 Clinical outcomes

The first time of assisted walking and eating and time of the removal of indwelling urinary and wound drainage catheters were significantly improved in the ERAS group (Table 3). The mean discharge time was significantly reduced from 7.67 ± 3.45 days in the traditional care group to 5.75 ± 2.46 days in the ERAS group (*P* < .001).

**Table 3:**
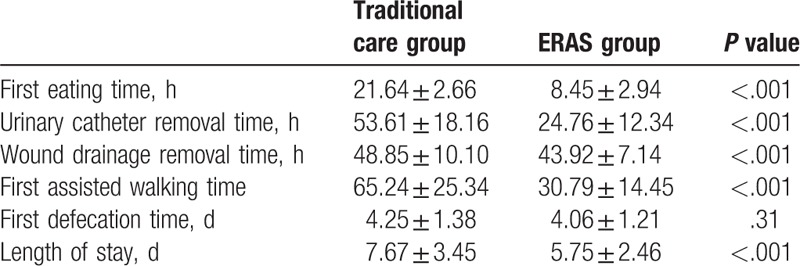
Postoperative physiological function outcomes.

Pain control was better in the ERAS group than traditional care group in terms of mean 3-day VAS score (2.72 ± 0.46 vs. 3.35 ± 0.46, *P* < .001) and mean 3-day maximum VAS score (3.76 ± 1.12 vs. 4.35 ± 1.15, *P* < .001) in 3 days after surgery (Table 4). There was no significant difference in the frequency of break-out pain between the 2 groups (42 vs. 30 times, *P* = .15).

**Table 4:**
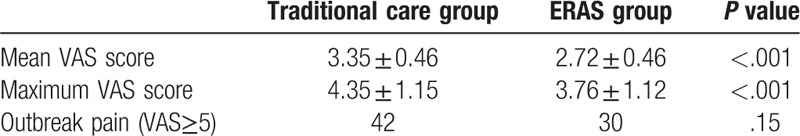
VAS pain score of 3-d after surgery.

The incidences of surgery-related complications are shown in Table 5. There was no perioperative mortality in the 2 groups. The morbidity rate was 21.05% (24 of 114 patients) in the ERAS group and 20.90% (23 of 110 patients) in the control group (*P* = .75). Wound infection occurred in 12 cases in the 2 groups with only 1 patient in the ERAS group who underwent debridement surgery. Early neurological deterioration after surgery due to epidural hematoma occurred in 1 patient in the ERAS group. In this case, neurological function was completely recovered by emergency surgery. Nausea and vomiting were more common in the ERAS group, probably due to the use of opiates. The low frequency of urinary infection in the ERAS group was probably attributed to the early removal of urinary catheters.

**Table 5:**
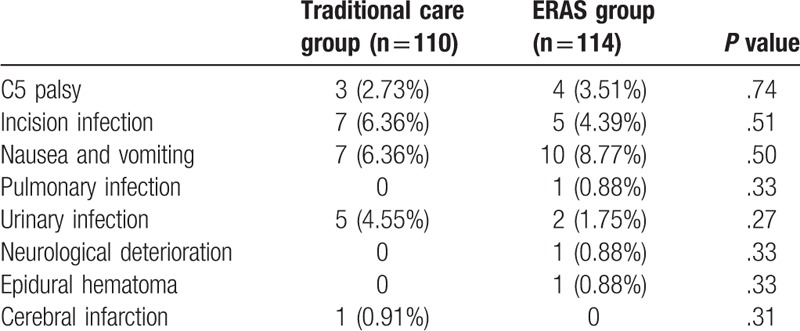
Postoperative complications and adverse reactions in detail.

### 3.3 Compliance with ERAS core elements

Compliance with the core elements of the perioperative ERAS protocol in the ERAS group is shown in Table 6. During the perioperative period, no patient underwent bowel preparation and the time of fasting prior to surgery was shortened, which had not been applied in the control group. Also, compliance with scheduled local intravenous analgesia, oral analgesia, and PONV prophylaxis was very high. Because the adverse effects of opioid drugs, such as respiratory depression and gastrointestinal adverse reactions, are worrisome, compliance with the use of an analgesia infusion pump was low (62.28%) in the ERAS group. In patients who did not use an analgesia infusion pump, oral tramadol was given when necessary. Compliance with early removal of the urinary and wound drainage catheters in the ERAS group were 86.84% and 92.11%. Removal of the urinary catheter was delayed in patients who developed dysuria preoperatively. The most common cause of delayed removal of the wound drainage catheter was a high drainage volume on POD 2, which may increase the risk of postoperative hematoma and infection. Compliance with an early regular diet and intermittent pneumatic compression were 98.25% and 95.61%. One hundred four patients (91.22%) performed bedside sitting and assisted walking on POD 1, while those with severe weakness of the lower limbs were unable to walk on POD 1.

**Table 6:**
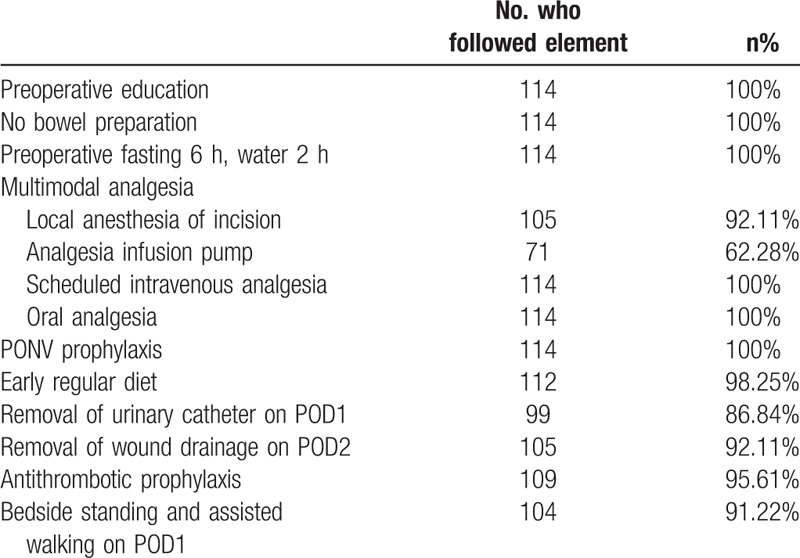
Compliance with ERAS core elements of the ERAS group.

## 4 Discussion

The ERAS protocol is an evidence-based multimodal perioperative care approach to reduce the length of hospitalization, diminish surgery-related complications, and improve early rehabilitation. While there have been various ERAS guidelines developed for many types of procedures in most major surgical specialties,^[14–17]^ there is paucity of research examining the application of an ERAS protocol in major spinal surgery and guidelines for spinal surgery have not been established. While few studies have investigated the application of an ERAS protocol in lumbar surgery, none have reported the use of such a protocol in cervical surgery. In the present study, using the comprehensive ERAS protocol improved patient recovery, reduced the length of hospital stay, and improved postoperative recovery of patients after laminoplasty.

There is convincing evidence that preoperative education can reduce preoperative anxiety and postoperative pain, and improve overall satisfaction. For fear of those catastrophic complications of spinal surgery, such as neurological deterioration, paralysis and even death, patients may feel vulnerable and have significant perioperative anxiety, which has a negative influence on the severity of postoperative pain and functional recovery.^[18,19]^ Pretreatment of anxiety and depression before surgery results in a significant reduction in postoperative neck pain, improvement in the quality of recovery, as well as overall satisfaction with care.^[20–22]^ In a study of 175 patients undergoing spinal surgery, Lee et al^[18]^ demonstrated that 87% of the patients reported preoperative anxiety attributed to the fear of surgery and found that faith in the medical staff and the surgeon's explanation of the procedure were the most helpful factors in overcoming preoperative anxiety. Preoperative education about the details of the operation, pain coping strategies, estimated hospital stay, and details of recovery may significantly change catastrophic thinking by patients of pain and risk, and thus avoid unnecessary stress.^[23,24]^ Preoperative expectation could influence the length of postoperative hospitalization. A randomized controlled trial of lumbar discectomy noted that the patients admitted as inpatients failed to be discharged as day cases, but not those admitted as outpatients.^[25]^ Moreover, there is an old Chinese proverb that stated “injury of muscles and bones takes 100 days to recover,” which is deeply rooted in Chinese minds. Therefore, Chinese people prefer to remain in the hospital until pain from bone fracture and surgical treatment is mostly relieved, which runs counter to the ERAS protocol. Thus, preoperative education to set reasonable expectations and rectify traditional ideas is important.

The type and extent of pain experienced by patients undergoing spinal surgery differ from those of other orthopedic patients. The reported positive effect of preoperative education on pain control in spinal surgery is encouraging. Papanastassiou et al^[26]^ reported that in a group of spinal surgery patients, those who attended a precare class reported better satisfaction with pain control (96% vs 83%), as compared with those who did not attend. In major spinal surgery for adolescent idiopathic scoliosis, individuals who received coping strategies for managing postoperative pain reported significantly less pain than the control group.^[27]^ In a case series of lumbar radiculopathy, immediately after preoperative education, patients scheduled for lumbar surgery achieved a significant reduction in postoperative pain and positive shifts in their beliefs about lumbar surgery,^[23]^ and these favorable behavioral changes continued throughout the 3-year follow-up period.^[24]^ In our ERAS group, preoperative education introduced the aim and procedure of the ERAS protocol, pain coping strategies and details of the operation. Pain control was better in the ERAS group than traditional care group in terms of mean VAS score and mean maximum VAS score in 3 days after surgery.

In the ERAS group, no bowel preparation was performed and fasting included cessation of liquids for 2 hours, as well as solid foods for 6 hours before anesthesia. Recent studies found that no preoperative bowel preparation is safe and associated with reduced postoperative morbidity in abdominal surgery.^[28,29]^ In posterior spinal instrumented fusion surgery, Olsen et al^[30]^ found no benefit, but rather a possible negative effect, from bowel preparation on gastrointestinal function; as omitting enema resulted in earlier postoperative defecation. Reducing the time of fasting preoperatively is meant to decrease the feeling of anxiety, hunger, and starvation-induced insulin resistance. Most patients in the ERAS group were able to drink fluids within 2 hours after surgery and consume a liquid diet at 6 hours. Daily fluid administration was restricted to 1000 mL according to the loss of blood and overall condition of the patient. It is beneficial to reduce postoperative respiratory and cardiovascular complications, and shorten the length of hospitalization.^[31,32]^

Patients undergoing major spinal surgery may easily become malnourished during the hospital stay, especially after reconstructive surgery for spinal scoliosis.^[33]^ Previous studies have reported that the benefits of early feeding include faster recovery of bowel function, lower rates of infectious complications, shorter hospital stay, and higher satisfaction.^[34,35]^ In the ERAS group, intake of oral fluids and solids offered after anesthesia awareness to secure energy intake was shown to be not associated with gastrointestinal adverse reactions, such as vomiting, abdominal distension, and constipation. The higher incidence of nausea and vomiting in the ERAS group may be attributed to the enhanced analgesic program, which consisted of the use of opioid drugs. Thus, the application of antivomiting drugs was regularly prescribed in the ERAS group and the infusion volume was restricted to 1000 mL/d.

The benefits of early postoperative activities are obvious. Getting out of bed “early” reduces the length of hospitalization and the incidence of perioperative complications, such as deep venous thrombosis, pulmonary embolism, pneumonia, atelectasis, urinary tract infections, sepsis, myocardial infarction, and stroke, among others.^[36,37]^ For patients who underwent lumbar surgery, an intense rehabilitation program under the instruction of a physiotherapist started on the day of surgery had significantly improved patient satisfaction and shortened the length of hospitalization without increasing the risks of complications and pain.^[38]^ For elderly patients aged > 65 years undergoing elective spinal surgery for correction of adult degenerative scoliosis, early ambulation within 24 hours after surgery significantly reduces the incidence of perioperative complications, shortens the duration of hospitalization, and contributes to improved perioperative functional status.^[39]^ They concluded that a delay in ambulation of only 24 hours was associated with higher complication rates and inferior functional outcomes. In the ERAS group, patients were encouraged to perform bedside standing and assisted walking on POD 1 with the help of a caregiver to avoid falling. Mannitol was given to our patients for 2 days to avoid wound swelling. The compliance of bedside standing and assisted walking on POD1 was low (90.43%), which is attributed to severe weakness of the lower limbs in some patients and delayed removal of wound drainage catheters. For those patients, on-bed movement is carried out under the guidance of a caregiver.

There was no significant difference in complication rates between the 2 groups, probably because of the small sample size and the poor compliance of certain elements of the ERAS protocol, suggesting that compliance may affect the observed outcomes. A report of more than 900 patients with colorectal cancer found that, compliance of greater than 70% with ERAS interventions lowered the risk of 5-year cancer-specific death by 42%, as compared to compliance of less than 70%.^[40]^ Therefore, doctors, nurses, anesthesiologists, and caregivers require ongoing education and training to implement ERAS protocols more strictly and cooperatively. Support by hospital management and the use of audits are also important, and long-term follow-up is needed to further evaluate the benefits of ERAS protocols. Another limitation of the current study is that the 2 groups of the patients were retrospectively selected from 2 time periods. That might lead to a possible biased result because our surgeons could have improved their skill during the 2 years.

## 5 Conclusion

The ERAS protocol is both safe and feasible for patients undergoing laminoplasty, and can decrease the length of postoperative hospitalization without increasing the risk of complications. Ongoing education and training should be given to multidisciplinary team including doctors, nurses, anesthesiologist, and carer to implement ERAS protocols more strictly and cooperatively. Long-term follow-up is needed to further evaluate the benefits of ERAS protocols.

## Acknowledgment

The authors sincerely acknowledge Dr Xin-lei Hu for his useful assistance in statistical analysis.

## Author contributions

**Conceptualization:** Zheng-kuan Xv, Jian Wang.

**Data curation:** Hao Li, Qun-fei Yu, Ying Ren.

**Funding acquisition:** Jun Li, Jian Wang, Qi-xin Chen.

**Investigation:** Hao Li.

**Methodology:** Ying Ren, Qi-xin Chen.

**Project administration:** Fang-cai Li, Ying Ren, Qi-xin Chen.

**Resources:** Zheng-kuan Xv, Qun-fei Yu.

**Software:** Gang Chen.

**Supervision:** Zheng-kuan Xv, Gang Chen.

**Validation:** Gang Chen.

**Writing – original draft:** Jun Li.

**Writing – review & editing:** Hao Li, Jian Wang, Fang-cai Li, Qi-xin Chen.
